# Characterization of complex New Kingdom funerary resinous mixtures uncovers liquorice-derived substances and pine nut oil

**DOI:** 10.1038/s41598-026-55926-7

**Published:** 2026-06-11

**Authors:** Martina Magni, Susanne Töpfer, Valentina Turina, Giulia Pallottini, Marco Samadelli, Frank Maixner, Albert Zink, Peter Robatscher, Alice Paladin

**Affiliations:** 1 Laboratory of Flavours and Metabolites, Laimburg Research Centre, Ora/Auer, Bolzano, 39040 Italy; 2 Fondazione Museo delle Antichità Egizie di Torino, Turin, 10123 Italy; 3 Institute for Mummy Studies, Eurac Research, Bolzano, 39100 Italy

**Keywords:** GC-MS, LC-HRMS, Ancient Egypt, embalming, bitumen, coffin, Biochemistry, Chemistry

## Abstract

Ancient Egyptian embalming substances and organic coatings applied to coffins and burial furnishings served both sacred and functional roles. However, their molecular characterization remains incomplete despite advances in analytical chemistry, highlighting the need for deeper approaches to refine compositional frameworks. This study investigates organic residues from the New Kingdom (2nd millennium BCE) funerary assemblage of Kha (outer bandages and middle and inner coffins), Merit (inner coffin), and a coeval little jar, all housed at the Museo Egizio in Turin, Italy. Gas chromatography-mass spectrometry (GC-MS) and liquid chromatography-high resolution mass spectrometry (LC-HRMS) were combined to determine whether the substances were bituminous or resinous and to detail their composition. The analyses revealed resinous compounds, with no detectable evidence of bitumen, and a diverse organic matrix dominated by plant-derived biomarkers. Distinct molecular profiles emerged between body-related materials and coffin coatings, supporting function-specific formulations. Notably, 18-β-glycyrrhetinic acid (liquorice-derived compound) and pinolenic acid (pine nut oil marker) were assigned, providing the earliest molecular evidence to date for the use of liquorice in a New Kingdom funerary context. Overall, these findings reveal function-related funerary preparations and selective ingredient use, underscoring the potential of integrated HRMS workflows to detect unconventional markers and advance the study on ritual practices.

**Supplementary Information:**

The online version contains supplementary material available at 10.1038/s41598-026-55926-7.

## Introduction

Recent advancements in analytical chemistry applied to archaeological science have significantly enhanced our understanding of the complex organic mixtures used in Ancient Egyptian funerary embalming practices. Analyses have shown that these archaeological materials contained a range of organic compounds^1–4^, including plant resins (e.g., *Pistacia* spp.), beeswax, animal and/or vegetable fats, and, in some instances, additives such as charcoal^5^ or bitumen^6,7^, used to achieve a ritualized black coloration. Such organic preparations served both sacred and practical functions within Egyptian funerary rites and religious ideology, where both the body and its coffin were treated as ‘vessels’ of rebirth and divine transition^8^. Therefore, the application of aromatic resins and oils extended beyond mere antiseptic utility^9^; they were integral to the religious logic of the embalming ritual, used to ritually purify, sanctify, and symbolically transform the deceased into an Osirian form^8^. Thus, a deliberate and codified use of materials was established not only for embalming and preserving the body, but also for preparing, treating, and decorating the coffin and burial furnishings. Black resinous coatings or fragrant balms, for instance, may have served to extend ritual protection to the material container of the body. Moreover, the substances were deliberately selected and combined to achieve specific olfactory and tactile properties^4^. Visual effects with symbolic and ritual significance, such as the use of bitumen and charred resins to produce a glossy black coating on coffins, may have been intended to evoke the regenerative power of Osiris and the fertile Nile silt, thereby embedding the deceased within a cosmologically resonant visual language^8^.

Despite major advances in analytical sensitivity and resolution^10^, comprehensive molecular characterization of archaeological organic residues remains challenging^2,11^. This is largely due to the intrinsic complexity of these matrices, which limits the extent to which routine analytical techniques can capture their full molecular diversity. However, multi-analytical strategies are commonly employed to enhance compositional insight^1,3^, as no single analytical platform currently provides exhaustive molecular coverage^10,12^.

The intact 18th Dynasty (New Kingdom, NK; 2nd mill. BCE) burial assemblage of the couple Kha and Merit, discovered by Ernesto Schiaparelli in 1906 in Theban Tomb TT8 at Deir el-Medina and housed at the Museo Egizio in Turin, provides an exceptional opportunity to investigate the molecular composition of funerary organic materials within a well-documented^13,14^ archaeological context (Suppl. Information Text S1). The assemblage included richly furnished grave goods and elaborately decorated coffins, consistent with the high status of Kha, an overseer of royal construction projects, and his wife Merit^15,16^.

Preliminary gas chromatography-mass spectrometry (GC-MS) analyses conducted in 2015 by Bianucci and colleagues^17^ on samples from the outer coffins and the external bandages of both Kha and Merit identified roasted *Pistacia* resin, balsam, and cedar oil, pointing to the use of valuable and ritually significant substances.

**Fig. 1:**
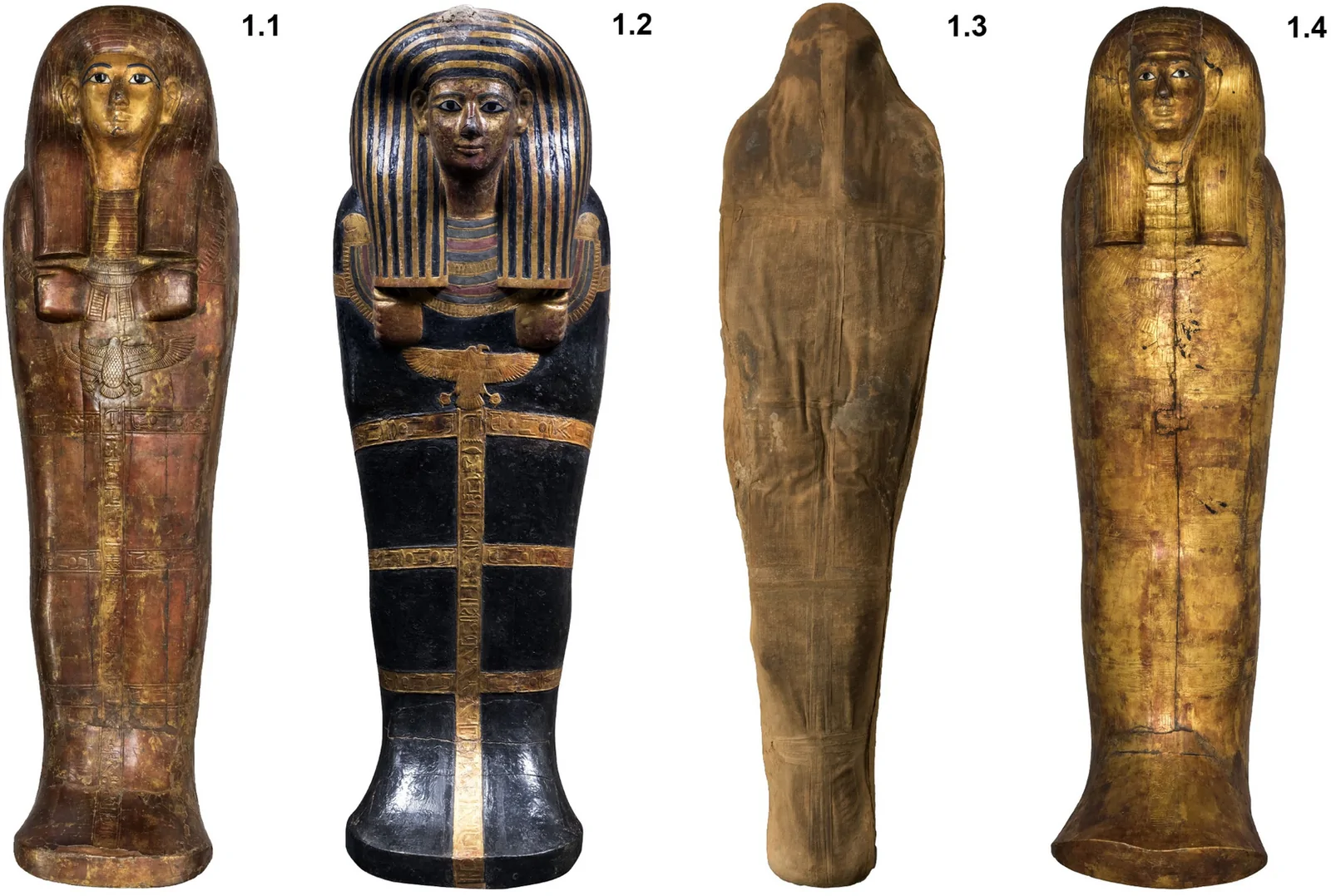
Coffins and outer textile of Kha, from the burial assemblage TT8 (Museo Egizio, Turin; see Supplementary Information, Text S1). 1.1: Inner coffin of Kha (Suppl.8429); 1.2: Middle coffin of Kha (Suppl.8316/01); 1.3: Outer bandages of Kha (Suppl.8431); 1.4: Inner coffin of Merit (Suppl.8470).

**Fig. 2:**
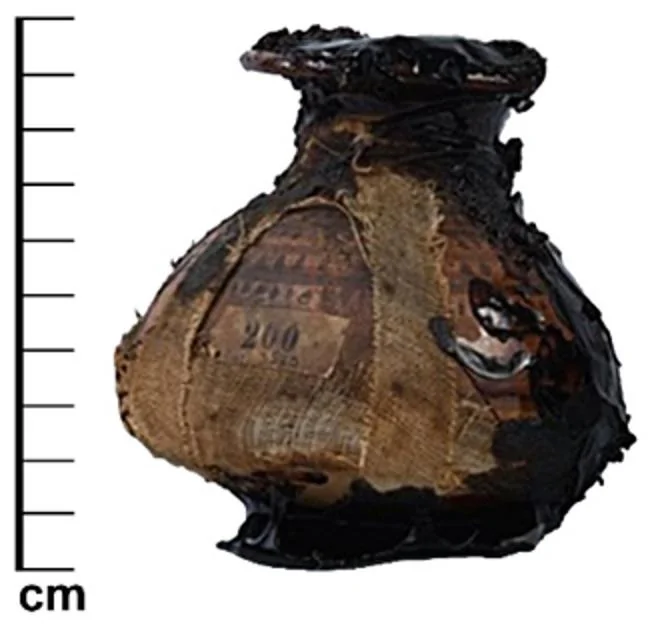
New Kingdom small jar (Cat. 3570) housed at the Museo Egizio in Turin. (Supplementary Information, Text S1, 1.5).

The present study expands upon previous investigations by analyzing a different set of organic residues from the same assemblage, including a newly sampled area Kha’s outer bandages, inner and middle coffins surfaces and the inner coffin of Merit (Fig. 1 and Suppl. Information Text S1). In addition, the organic content of a contemporaneous small jar (Fig. 2 and Suppl. Information Text S1), not associated with tomb TT8, was analysed to provide comparative insight into NK embalming practices^6,8^.

To overcome the analytical limitations inherent to complex archaeological matrices, we implemented an integrated analytical strategy combining GC-MS and liquid chromatography–high resolution mass spectrometry (LC-HRMS). An iterative data acquisition strategy coupled with automated data processing was employed to enhance detection of minor and degraded constituents, increase molecular coverage, and enable cross-platform validation of compound assignments.

Within this framework, the study aims to:

(i) determine whether the embalming substances were primarily resinous or bituminous in nature, and tentatively assign their botanical origins.
(ii) establish a comprehensive molecular characterization of the materials used in both embalming and coffin coating processes.

## Results

### GC-MS bitumen: analysis of non-polar compounds

The results of the typical markers (i.e., hopanes and steranes) of bitumen presence are shown in Table 1. A more comprehensive overview of the detected molecules is provided in Supplementary Table S1. GC-MS analysis revealed no detectable traces of bitumen in any of the five investigated samples.

**Table 1:** Subset of GC-MS data obtained with bitumen analysis approach. A more comprehensive overview of the detected molecules is provided in Supplementary Table S1.

SAMPLE (museum inventory number)	ALKANES	
	Hopanes 191 m/z Steranes 217 m/z	Short and medium linear chain
Inner coffin of Kha *(Suppl.8429)*	n.d.	✔
Middle coffin of Kha *(Suppl.8316/01)*	n.d.	n.d.
Outer bandages of Kha *(Suppl.8431)*	n.d.	n.d.
Inner coffin of Merit *(Suppl.8470)*	n.d.	✔
New Kingdom jar *(Cat.3570)*	n.d.	✔

✔= compound or compound class identified in the sample.

n.d.= not detected.

### GC-MS lipid: analysis of polar compounds

**Fig. 3:**
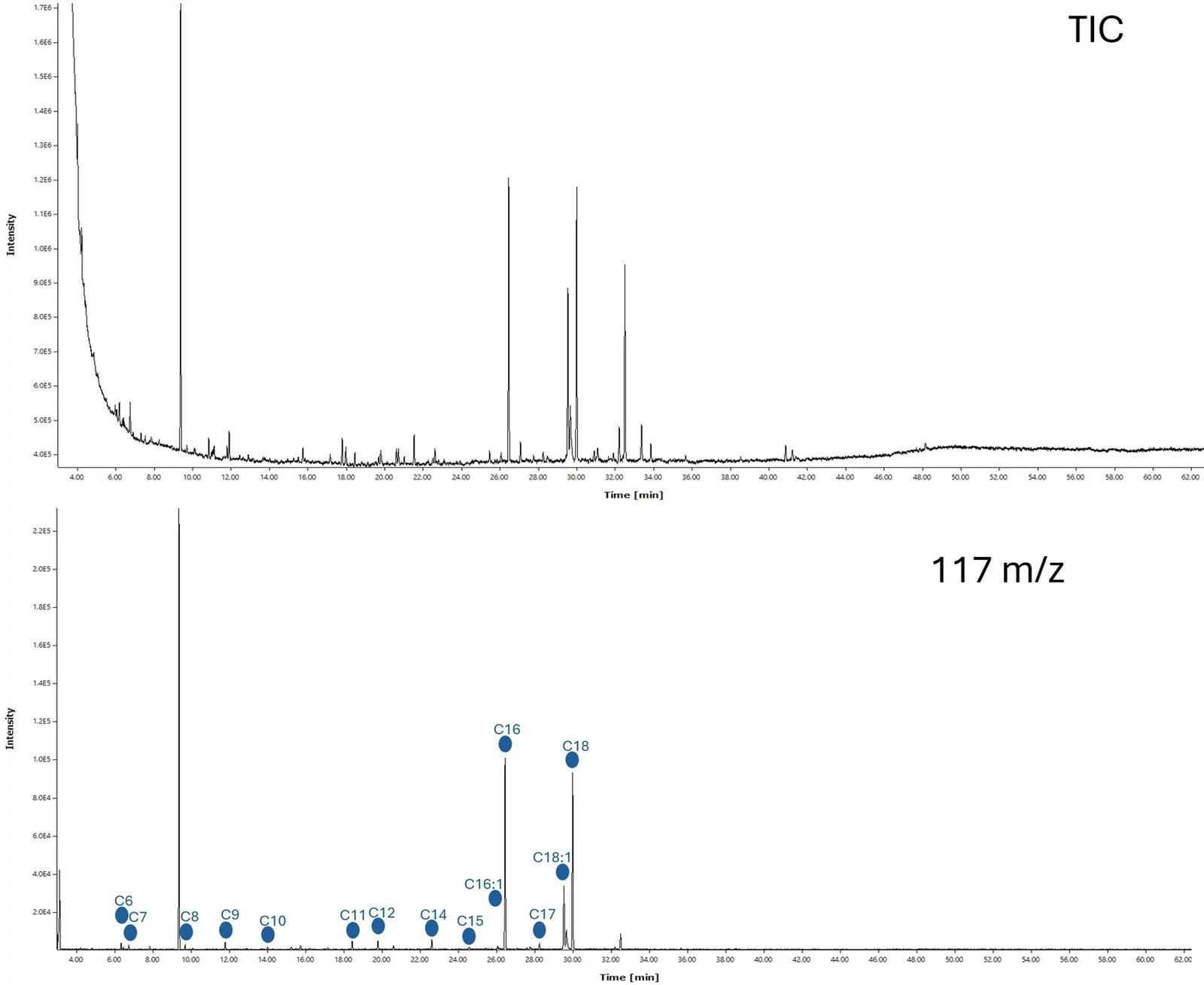
GC-MS analysis of the lipidic fraction of middle coffin of Kha (Suppl.8316/01). In the upper part, the Total Ion Current (TIC) chromatogram is shown, and in the bottom part, the chromatogram of extracted ion 117 *m/z* that is a typical fragment of FA class.

Many classes of compounds were detected within the lipidic fraction submitted to GC-MS characterization (Table 2). Fatty Acids (FAs) and α,ω-carboxylic acids are present in all evaluated samples. As an example, Fig. 3 reports the chromatographic profile of FAs detected in the sample from the middle coffin of Kha (Suppl.8316/01). Some alcohols with short carbon chain were detected in the sample collected from the NK small jar (Cat.3570) and from the outer bandages of Kha (Suppl.8431). The presence of benzoic acids and derivatives, like vanillic acid, was found in all samples, except in the NK jar. Detected alkanes were linear, branched and showed a maximum number of 16 carbon atoms. Terpenoids were detected in all the coffin samples (Table 2 and Suppl. Table S2). Detection of three monosaccharides supports a plant-based recipe of the balm covering the outer bandages of Kha (Suppl.8316/02).

**Table 2:** Subset of GC-MS data obtained with lipid analysis approach. Refer to Supplementary Table S2 for the complete list of all the identified compounds.

SAMPLE (museum inventory number)	FATTY ACIDS		α,ω-CARBOXYLIC ACIDS	HYDROXY-BENZOIC ACIDS	TERPENOIDS		SUGARS
	Even C_*n*°_ Odd C_*n*°_ Short chain FA	Long chain FA		Hydroxybenzoic ac. Vanillic ac.	di-	tri-	
Inner coffin of Kha *(Suppl.8429)*	✔	n.d.	✔	✔	n.d.	✔	n.d.
Middle coffin of Kha *(Suppl.8316/01)*	✔	n.d.	✔	✔	✔	✔	n.d.
Outer bandages of Kha *(Suppl.8431)*	✔	✔	✔	✔	n.d.	n.d.	✔
Inner coffin of Merit *(Suppl.8470)*	✔	n.d.	✔	✔	n.d.	n.d.	n.d.
New Kingdom jar *(Cat.3570)*	✔	✔	✔	n.d.	n.d.	n.d.	n.d.
**Marker-related** **possible sources**	Vegetable/animal fat or oil^18,19^		FA oxidation products^19^	Aromatic plants/ gums^20,21^	Coniferous resin^22^	Pistacia/ Boswellia^23^	Plant gum^24^

✔= compound or compound class identified in the sample; n.d.= not detected; FA= Fatty Acids; C_n°_= Number of carbons.

### LC-HRMS: molecular profiling focused on lipids

**Fig. 4:**
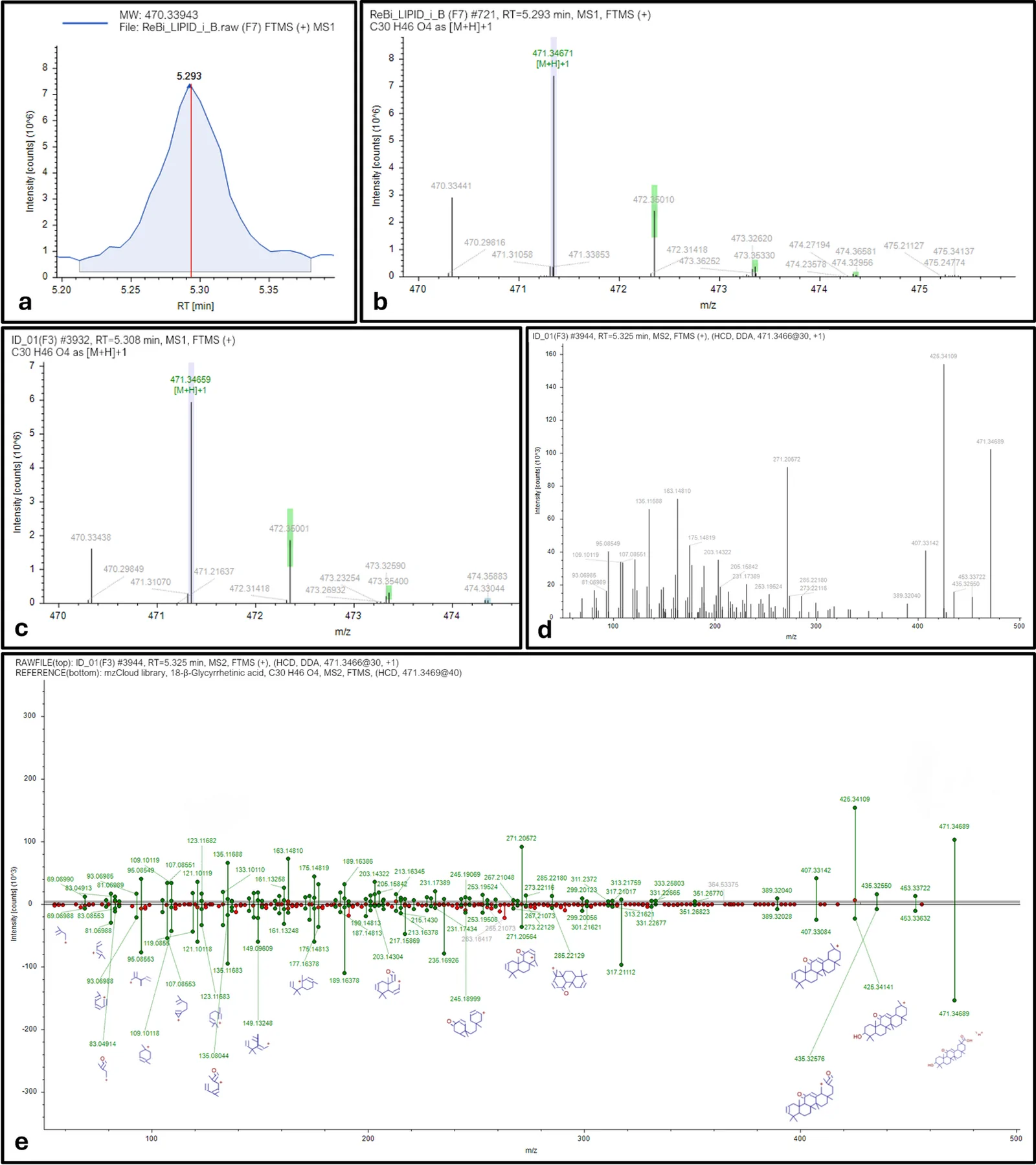
Mass spectrometry data acquired in positive ionization mode, used for the match in CD3.3 of 18-β-glycyrrhetinic acid (18-β-GA) for sample Suppl.8316/01. For spectra obtained in negative ionization mode, refer to Suppl. Information Fig. S1. **(a)** Extracted ion chromatogram of MW (Molecular Weight) 470.33943, with chromatographic peak of 18-β-GA at RT = 5.293 min; **(b)** MS1 spectrum of m/z 471.34671 at RT = 5.293 min; **(c)** m/z 471.34659 peak of the associated ID1 scan aligned with (a); **(d)** DDA spectra of parent ion m/z 471.3466@30; **(e)** mirror plot comparing experimental MS2 data acquired and library MS2 from mzCloud.

Table 3 presents a summary of the data (Suppl. Tables S2 and S3) putatively obtained through an untargeted lipidomic approach (Suppl. Table S4), as annotated compounds. The molecule 7-oxodehydroabietic acid was detected in all the samples, with the exception of the NK jar (Cat.3570), in which it was not found. The second most widely present marker is 18-β-glycyrrhetinic acid (18-β-GA), detected in all coffin samples both in positive and negative polarity mode. Solely for the middle coffin of Kha (Suppl.8316/01) it was observed along with its oxidation product: 3-oxo-glycyrrhetic acid (3-oxo-GA). Figure 4 presents the data used for the match of 18-β-GA in one of the analysed samples such as the middle coffin of Kha (Suppl.8316/01). In particular, Fig. 4a shows the chromatographic peak of monoisotopic mass 470.33943, present at retention time (RT) = 5.293 min. In Fig. 4b, the MS1 spectrum is reported at the same RT with the specific focus on the molecular ion, highlighted in lavender, as the A_0_ isotope, [M + H]^+^= 471.34671. Three isotopic peaks (A_1_= 472.35010, A_2_= 473.35330 and A_3_= 474.32956), highlighted in green, exhibit the expected relative intensities and spectral distance with respect to A₀. These findings confirm the validity of the proposed molecular formula within the defined analytical tolerances.

Same spectrum (Fig. 4c) is obtained at the same RT for the run of the first identification analysis (ID; refer to the Materials section for more information on the ID files) where there are the peaks A_1_ and A_2_ highlighted in green and A_3_ in light blue because a robust check is not possible due to the low intensity, nearly at the noise level. Figure 4d reported the fragmentation spectra of the A_0_ ion detected in first ID run: 471.3466 m/z undergone fragmentation in the collision cell. In Fig. 4e, the mirror plot presents a comparison between the experimental spectra obtained by data-dependent analysis (DDA) and the reference spectrum stored in the mzCloud database.

Moreover, markers indicating the resinous nature of the samples were found in the coffins and in Kha’s outer bandages (Table 3, Suppl. Tables S2 and S3). A broad range of FAs was detected in the small jar (Cat.3570) and in the outer bandages of Kha (Suppl.8431). Evidence for the presence of frankincense derived from *Boswellia sacra* is provided by the detection of boswellic acid, although this compound was identified only in Kha’s inner coffin (Suppl.8429).

**Table 3:** Subset of LC-HRMS data obtained with lipid analysis approach. Refer to Supplementary Table S3 for a complete list of annotated compounds.

SAMPLE (museum inventory number)	ANNOTATED COMPOUNDS							
	7-Oxodehydroabietic Acid	18-β-Glycyrrhetinic / 3-Oxoglycyrrhetic Acid	Oleanolic Acid	Moronic / Masticadienoic Acid	Betulonal	Boswellic Acid	Fatty Acids	Mucronine A / Nootkatone / Shogaol / Turmerone
Inner coffin of Kha *(Suppl.8429)*	✔	✔(+/-)	✔	n.d.	✔	✔	n.d.	✔ (T, N)
Middle coffin of Kha *(Suppl.8316/01)*	✔	✔(+/-)	✔	✔	n.d.	n.d.	n.d.	✔ (N)
Outer bandages of Kha *(Suppl.8431)*	✔	n.d.	n.d.	n.d.	n.d.	n.d.	✔	✔ (M, S)
Inner coffin of Merit *(Suppl.8470)*	✔	✔ (+/-)	n.d.	✔	✔	n.d.	n.d.	✔ (T, N)
New Kingdom jar *(Cat.3570)*	n.d.	n.d.	n.d.	n.d.	n.d.	n.d.	✔	n.d.
**Marker-related** **possible source**	Coniferous resin^25^	Liquorice^26^	Pistacia resin^25,27^	Mastic resin^25,28^	Dipterocarpaceae resins^29^	*Boswellia* sacra^30^	Vegetable/ Animal fat^18,19^	Spices^31–34^

✔= compound or compound class detected in the sample. n.d.= not detected.

(+/-)= detected both in positive and negative ionization mode.

(M)= Mucronine A; (N)= Nootkatone; (S)= Shogaol; (T)= Turmerone.

## Discussion

The first goal of the project was to determine, through GC–MS analysis, whether the selected samples were predominantly bituminous or resinous. The use of bitumen in NK embalming was not uniform but appears to have varied according to the funerary material examined. For example, bitumen has been identified in balms from a NK canopic jar containing the mummified lungs of Senetnay^2^, suggesting a potential targeted application linked to contextual or symbolic considerations. The absence of hopane or sterane^6,35,36^, along with the presence of only alkanes with short chain length (C_9_-C_16_) and a linear structure, indicates that no detectable traces of bitumen were present in all the five samples analysed. This finding agrees with Bianucci and colleagues^17^ who reported the absence of bitumen in the outer bandages of both the mummified individuals of Kha and Merit, and in their outer coffins. In the present study, neither the outer bandages of Merit nor the outer coffins were analysed; moreover, although both studies include a sample from Kha’s outer bandages, the specimen examined here derives from a different sampling location (Suppl. Information Text S1).

The second goal was to characterize the resin-based mixtures through a synergistic approach integrating GC-MS with LC-HRMS, with the aim of maximizing the coverage of the samples’ chemical space. In LC-HRMS, software-mediated acquisition was employed to automate the generation of dynamic inclusion and exclusion lists, thereby optimizing DDA. This approach significantly increased both the depth of compound coverage and the confidence in spectral characterization compared to conventional methods lacking software-assisted acquisition.

Unlike previous investigations^2,37^ on NK balm samples, beeswax was not detected in any of the specimens selected for this research. In fact, none of the molecular markers, such as long carbon chain FA (C_22_-C_30_), fatty alcohols, and palmitate esters^22,38^, were found. Once again, these results are consistent with those reported by Bianucci and colleagues^17^, who documented the absence of beeswax in the outer coffins (not included in the present work) of Kha and Merit. Beeswax was reported in the outer bandages of Merit (not investigated here) but not in those of Kha; the latter observation aligns with the results of the present study.

Lipids have previously been documented in resinous substances used in NK funerary goods^9,37^. The present investigation reinforces this lipidic signature through the proposing of multiple diagnostic molecular markers. Free fatty acids (FAs) were detected in all five samples analysed (Table 2; Suppl. Tables S2 and S3), likely resulting from the degradation of triglycerides, the main constituents of fats and oils. These findings support the use of oils or fats as a component of the embalming materials and coatings applied to the coffin surfaces. Only trace amounts of monoglycerides were observed in all samples, except for the inner coffin of Kha, via GC–MS analysis, and no di- or triglycerides were detected, likely due to the archaeological nature of the specimens and the preservation conditions, which led to extensive molecular degradation. Additional insights include the detection of odd-chain monocarboxylic fatty acids (mainly C_15:0_ and C_17:0_), which may indicate an animal origin, although bacterial degradation cannot be excluded as a contributing factor^39^. However, no animal sterols such as cholesterol were observed. Moreover, the length of the carbon chains, particularly those over 20 carbons, generally indicates a plant origin rather than an animal one. Therefore, the hypothesis of vegetal provenience of FAs is further supported by the presence of ricinoleic acid (12-hydroxyoctadec-9-enoic acid), a typical marker of castor oil^18,19^, which was annotated by LC-HRMS in the small NK jar and in the bandages of Kha. Another type of FAs, present in all the samples, were α,ω-dicarboxylic acids, which are oxidation products of their monocarboxylic FAs precursors. This oxidative transformation typically occurs at the site of unsaturation (double bond), reflecting degradation pathways consistent with ancient organic residues. Reasonably, the originally present oleic acid was oxidized through a chain break at the unsaturated C_9_, resulting in the formation of azelaic acid, a well-documented degradation product^19,40^. Hydroxylated fatty acids (hydroxy-FAs) were also counted among the oxidation products derived from lipid molecules. The same hydroxy-FAs were detected by both GC-MS and LC-HRMS in samples from the small jar and Kha’s bandages. Specifically, the presence of 9,10-dihydroxy-octadecanoic acid, a known oxidation product^41^, further confirms the original presence of oleic acid. Based on the molecular profiles (Suppl. Table S3), the jar content shows a shared lipidic core with the substance found on the bandages (e.g., ricinoleic and dihydroxystearic acids). It is therefore plausible to hypothesize that the jar originally contained a lipid-based precursor—likely a vegetable oil mixture— potentially related to preparatory or storage vessel. However, the exact ritual or practical function cannot be firmly established based on the available evidence. Moreover, determining the potential sources of oil or fat remains challenging, as the fatty acid profiles do not clearly point to a single origin and likely reflect a mixture of substances, many of which share common fatty acid constituents, further complicating source attribution.

Hydroxy-benzoic acids (Table 2) likely originate from plants^42,43^, and may be associated with the use of scented materials and aromatic resins. In addition, the effectiveness of the synergistic application of the employed techniques is demostrated by the detection of a typical marker of plant resin such as 7-oxodehydroabietic acid identified by LC-HRMS, and/or its unoxidized counterpart, dehydroabietic acid, by GC-MS. Those markers of plant resin were found in four out of five samples (excluding the jar). These compounds are derived from the ancient resin of the coniferous^21,25^. The Papyrus Ebers^44^ references conifer resin multiple times, describing it in various recipes combined with plant- or animal-based ingredients, applied beneath textiles, grounded, or cooked together to produce resinous remedies. Furthermore, conifer oil, one of the seven sacred oils known since the Old Kingdom, was used among other purposes in embalming rituals^8^.

Several specific biomolecular markers of spices were also tentatively assigned in the studied samples, except in the jar, including shogaol from *Zingiber officinale*^32^, turmerone from *Curcuma longa*^31,45^, (+)-nootkatone from *Cupressaceae/Citrus*^33,46^, and mucronine A from *Ziziphus mucronata*^34^. These spices may have been incorporated into the recipes for their pleasant scent or antimicrobial properties^47,48^.

Sugars were also present in Kha’s outer bandages, as also documented by Bianucci and colleagues^17^. Furthermore, the detection of both furanose and pyranose ring forms supports the hypothesis that a plant gum was intentionally added as an ingredient. This interpretation is favored over the alternative explanation that these sugars originated from the cellulose-based textile itself, as proposed by Buckley and colleagues^24^.

A noteworthy result of this study is the putative presence of 18-β-glycyrrhetinic acid, a characteristic biomarker of the *Glycyrrhiza* genus (e.g., *Glycyrrhiza glabra*, *G. uralensis*, *G. inflata*), a wild leguminous plant commonly known as liquorice (Suppl. Information Text S2). This plant is native to Asia and the Mediterranean area, it was known also by the Greeks, and it has been widely used in Chinese medicine for its anti-inflammatory, antioxidant, and antimicrobial properties^49–51^. Not previously reported in NK funerary specimens, this compound shows a match for all the coffins examined in the present study (Suppl. Information Text S1). Notably, both 18-β-glycyrrhetinic acid and its oxidized derivative, 3-oxo-glycyrrhetinic acid, were detected exclusively in the sample from Kha’s middle coffin, as presented in Table 3 and Supplementary Table S3. This finding represents a new contribution to the chemical characterization of NK coffins coating and suggests a potentially unique use of liquorice-derived substances in this context. Liquorice has recently been mentioned in molecular studies, such as in the work by Tanasi and colleagues^3^ on organic residues sampled from a Bes-vase dated to the Ptolemaic–Roman period. Its detection in an earlier funerary context, as revealed by the present study, underscores the significance of this discovery and opens new avenues for understanding the diversity of ingredients employed in NK funerary practices. However, in the absence of textual, linguistic, or iconographic evidence, the historical use of liquorice in Ancient Egypt remains unconfirmed (Suppl. Information Text S2). Claims of its presence in tombs such as that of Tutankhamun^52,53^ or in medical papyri^44^ appear to stem from modern misinterpretations or the uncritical repetition of secondary sources. Therefore, LC-HRMS analysis was also conducted in negative ionization mode (Suppl. Information Fig. S1), which is particularly effective for enhancing the detection of acidic compounds, thereby further supporting the spectral matching of this molecular finding. While these results provide compelling support for this annotation, future research involving the injection of pure analytical standards, sample fractionation, purification, and NMR-based structural characterization, together with semi-quantitative investigations, will further strengthen and contextualize this finding within the framework of ancient Egyptian embalming practices.

A second novel compound putatively annotated in this work through LC-HRMS analysis is pinolenic acid, a molecular marker of pine nut oil, detected in the sample collected from Kha’s outer bandages. Enabled by the high sensitivity and mass accuracy of LC-HRMS, the detection of pinolenic acid expands the molecular profile of the sample beyond that reported by Bianucci et al.^17^. While pinolenic acid has been documented in materials dated between the 4th − 3rd century BCE^3^, to the Authors’ knowledge, it has not been documented in any studies of NK specimens.

In conclusion, the analysed organic materials were examined using a multi-analytical approach, which revealed no detectable evidence of bitumen or beeswax. The small jar content and the organic substance that covers the outer bandages of Kha exhibit a shared lipidic base, likely of vegetal origin and characterized by the presence of castor oil biomarker, suggesting an oil-based component likely involved in embalming preparation. In contrast, the coffin coatings are distinguished by a marked prevalence of conifer-derived resins and aromatic plant (e.g., Pistacia, Conifer, *Boswellia sacra*) compounds. Moreover, the presence of 18-β-glycyrrhetinic acid in all coffin samples represents the earliest molecular evidence to date of liquorice-derived substances in a NK funerary context. Likewise, pinolenic acid assignment in Kha’s outer bandages has not previously been documented in materials from this period. Overall, these findings indicate function-related (body treatment vs. coffin coatings) preparations and selective ingredient use within the funerary sphere, underscoring the value of integrated high resolution-mass spectrometry analysis for reconstructing ancient ritual practices.

## Methods

Samples were collected at the Museo Egizio in Turin (Italy), from five distinct locations: the outer dark layer observable on some of the bandages on the mummified individual Kha, the middle and inner coffins of Kha, and the inner coffin of his wife, Merit (Fig. 1). An additional sample, contemporaneous with Kha and Merit, but not directly associated with their burial assemblage, was obtained from a jar of unknown provenance (Fig. 2). Sampling was authorized by the Museo Egizio and carried out according to standardized European procedures for cultural heritage materials (Supp. Information Text S1). Prior to sampling, museum documentation and consultations with the curatorial staff confirmed that no modern consolidants or solvent-based conservation treatments had been applied to the archaeological remains. Detailed information on the sampling locations and sample typology are provided in the Supplementary Information, Text S1. The collected materials were then stored at the Anthropology laboratory of the Institute for Mummy Studies of Eurac Research (Bolzano, Italy). They were then sealed in 1 mL Eppi^®^ tubes and maintained under the same temperature conditions as those of the museum environment^54^. Owing to the minimally invasive sampling approach, required by the uniqueness and conservation constraints of the artefacts, sample quantity was limited. Consequently, analytical replicates were not feasible for all samples; therefore, no replicates were performed (Suppl. Table S5).

For the analytical procedures, all solvents were LC–MS grade and purchased from VWR, while ultrapure water was obtained using an Integral 15 system (Millipore, Burlington, USA). The reagent BSTFA + 1% TMCS was purchased from ITW Reagents (P/N 3555881606).

### GC-MS bitumen: analysis of non-polar compounds

Following Hertzog et al.^1^, about 1 mg of the sample was weighed into a glass vial and covered with 1 mL heptane, then dipped for 5 min in ultrasonic bath (VWR Leuven) at room temperature; an aliquot was transferred into a GC glass vial and 1 µL was injected with splitless mode at 250 °C into GC system (GCMS-QP 2010) equipped with a column HP-5MS (30 m; 0,25 mm ID; 0,25 μm, Agilent Technologies). The interface temperature was 320 °C, the EI source set at 230 °C. The acquisition range was set between 30 and 700 *m/z (*Mass-to-charge ratio*)* for the Full Scan (FS) and at *m/z* 191 for hopanes and 217 *m/z* for steranes^35,36^, as selected ion monitoring (SIM). The oven programmed temperature was 60 °C for 2 min, 60–120 at 15 °C/min, held for 2 min and final ramp of 5 °C/min until 320 °C. Solvent blank and procedural blank were injected before and after samples. Peak assignment was based on the comparison with GCMS Solution software using the spectra library NIST17 version.

### GC-MS lipid: analysis of polar compounds

Following Hertzog et al.^1^, about 1 mg of sample was weighted into a glass vial and directly derivatized as follows: 100 µL of (BSTFA + 1% TMCS) and 10 µL of acetonitrile (ACN) at 75 °C for 1 h. The vial content was evaporated to dryness under a gentle stream of nitrogen, resuspended with 0.5 mL isooctane and injected with spitless mode at 250 °C into a GC system (GCMS-QP 2010) equipped with a column HP-5MS (30 m; 0,25 mm ID; 0,25 μm, Agilent Technologies). The interface temperature was 320 °C, the EI source was 230 °C and the acquisition range was set between 30 and 700 *m/z*. The oven programmed temperature was 60 °C for 2 min, 60–120 at 15 °C/min, hold for 2 min and final ramp of 5 °C/min until 320 °C. Solvent blank and procedural blank were injected before and after samples. Peak assignation was based on the comparison with GCMS Solution software using NIST17 library spectra.

### LC-HRMS: lipid focus molecular profiling

Based on established protocol^55^, each sample was extracted prior to analysis. Approximately 2 mg was weighed into a glass vial. To this, 700 µL of *t*-butyl-methyl-ether and methanol mixture (3:1) was added. For enhancement of homogeneous mixing, 30 s of vortex, 45 min of orbital shaker at 100 rpm and 15 min of iced ultrasound bath were applied, in sequence, to the vial. Next, 650 µL of water and methanol (3:1) was added, and the vial was vortexed for 1 min. The vial was then centrifuged at 2000 rpm, 4 °C for 5 min to separate the two layers. The upper layer, containing the lipid fraction, evaporated to dryness under a gentle stream of nitrogen and resuspended in 600 µL of ACN and 2-propanol (7:3) and injected (5 µL) into HPLC Vanquish Flex coupled with mass spectrometer Orbitrap Exploris 240 (Thermo Fisher Scientific). The mobile phase A consisted of water with 0.1% formic acid. Mobile phase B was a mixture of ACN and 2-propanol (7:3) with 0.1% formic acid. Therefore, the gradient was applied with a flow rate of 300 µL/min as follows: starting from 55% B, held for 1 min, then increased to 75% B over three minutes. In 8 min to 89% then straight to 100% within 3 min, where it was held for 5 min. This process was followed by a reconditioning step, with the initial conditions, for nine minutes. Chromatographic separation was achieved with Acclaim™ RSLC PA II column (150 mm x 2.1 mm x 2.2 μm particle size, Thermo Fisher Scientific), maintained at 60 °C. Atmospheric Pressure Chemical Ionization (APCI) source was set at 4 µA of positive discharge current, 400 °C of vaporizer temperature, with 30, 10 and 0 as sheath, auxiliary and sweep gas, respectively; the ion transfer tube was at 320 °C, the expected LC peak width was 8 s, the default charge state was 1, and AcquireX^56^ method modification was set as activated. Calibration of the mass accuracy was performed externally at less than 1 ppm (parts per million), and internally at Run Start for each run. Quality assurance procedure, also requested by the acquisition workflow, was to inject one solvent blank and two procedural blanks within every sequence. Each sample was submitted under AcquireX^56^ Deep Scan sequence (Suppl. Table S4), with default parameters, except for exclusion override = 5 and automatic adding of isotopes enabled. The exclusion list was created based on the third injection of the blank. The inclusion list with accurate mass precursors was populated based on a full scan of the lipid fraction extracted at 180,000 resolution (100 to 1300 *m/z*). Fragmentation spectra were acquired, based on the inclusion list, with four injections of the same sample with iterated scan (ID files): the MS1 lead scan was at 60,000 resolution and the related DDA scan was at 30,000 resolution, with a fixed intensity threshold as 1.00E4; targeted mass included or excluded had 5 ppm mass windows, and the HCD fragmentation was performed with absolute energy as 30 V. The RF lens was 70%, AGC target customized at 100% for the full scan and 70% for DDA and maximum injection time was automatic. A further test was also performed in negative mode, for all the samples collected from the coffins, with all the other parameters unchanged.

### Data analysis: compound discoverer

Raw files of blank, sample and all four ID files were submitted to the software CD3.3, using a default workflow for lipidomic called “Untargeted Lipidomics using Online Databases, LipidBlast in-silico library and LipidMaps database” with main modifications listed below. In the node select spectra the retention time upper limit was cut at 20 min; in the scan event filters HCD was selected as activation type; in the detect compounds node mass tolerance was set to 5 ppm, with a minimum peak intensity of 1.0E05, minimum number of scans per peak equal to 4, signal to noise detection as 1.5, remove baseline as true, ions [M + H]^+1^, [M + H-H_2_O]^+1^, [M + K]^+1^, [M + Na]^+1^, [M + ACN+H]^+1^, [2 M + ACN+H]^+1^, [2 M + H]^+1^, [2 M + H]^+2^. The molecular network node was cut from the workflow. Table 3 reported those analytes that comply with the following conditions: mzCloud^57^ score of at least 75 or match in ChemSpider^58^ or MassList databases (LipidMaps Structure Database). Other molecules match with CD3.3 workflow and relative tentative botanical sources are reported in Supplementary Table S3. For each feature, a natural source is proposed, where possible, based on information stored in online databases: ChEBI^59^, Human Metabolome^60^, LIPIDMAPS^61^, LOTUS^62^, PlantaeDB^63^, PlantFA^64^, PubChem^65^.

## Supplementary Information

Below is the link to the electronic supplementary material.

Supplementary Material 1

Supplementary Material 2

## Data Availability

All data generated or analysed during this study are included in this published article and in the Supplementary Information. The raw data that supports the findings are available from the corresponding author upon request.
