# Supplementary material for: Characterization of complex New Kingdom funerary resinous mixtures uncovers liquorice-derived substances and pine nut oil
Source: Sci Rep. 2026 Jun 11;16:26611. doi: 10.1038/s41598-026-55926-7 (PMC13503808; doi:10.1038/s41598-026-55926-7)
Supplement: Supplementary file 1 [file 41598_2026_55926_MOESM1_ESM.pdf]

Supplementary Table S1. Extended results for bitumen for all evaluated samples. ✓ indicate the presence of the molecules.

Chemical class	Carbon chain lenght	Annotated Compound	Inner coffin of Kha (Suppl.8429)	Middle coffin of Kha (Suppl. 8316/01)	Outer bandages of Kha (Suppl.8431)	Inner coffin of Merit (Suppl.8470)	New Kingdom jar (Cat. 3570)
ALCOHOLS	C5	1-Butanol, 3-methyl-			✓	✓	
	C7	3-Hexanol, 5-methyl-			✓		
	C8	1-Heptanol, 6-methyl-			✓		
	C8	1-Pentanol, 2-ethyl-4-methyl-				✓	
ALKANES	C9	Heptane, 3,3-dimethyl-	✓			✓	
	C10	Octane, 3,3-dimethyl-				✓	
	C21	5,5-Diethylheptadecane				✓	
CARBOXYLIC ACIDS and DERIVATIVES		Hexanoic acid, 3-hydroxy-, methyl ester			✓		
		Carbonic acid, heptyl prop-1-en-2-yl ester	✓		✓	✓	✓
		Methoxyacetic acid, heptyl ester			✓		
		Oxalic acid, heptyl propyl ester	✓			✓	✓
	C16	n-Hexadecanoic acid					✓
	C18	Octadecanoic acid	✓			✓	✓
DIALKYL ETHERS		1-Propene, 2-methyl-3-propoxy-	✓			✓	✓
		2-Heptene, 1-ethoxy-, (Z)-			✓		
		2-Hexanone, 5-methyl-					✓
EPOXIDES		Oxirane, 2-butyl-3-methyl-, cis-	✓		✓	✓	
		Oxirane, 2-ethyl-3-propyl-	✓			✓	✓
		Oxirane, 2-methyl-3-propyl-, trans-			✓		
		Oxirane, octyl-			✓		
Fatty acid esters		Heptanoic acid, 2-methyl-2-butyl ester				✓	
Fatty acid esters		Succinic acid, di(2,4-dimethylpent-3-yl) ester			✓		
Fatty amide		13-Docosenamide, (Z)-					✓
Lactones		7,9-Di-tert-butyl-1-oxaspiro(4,5)deca-6,9-diene-2,8-dione					✓
Ketones		2-Heptanone	✓		✓	✓	
Ketones		3-Heptanone			✓		
Organic nitrogen compounds		2-Propanamine, N-ethyl-N-nitroso-				✓	
Oxetanes		Oxetane, 2-methyl-4-propyl-	✓		✓	✓	
TETRAHYDROFURANS		Furan, 2-butyltetrahydro-	✓			✓	
		Tetrahydrofuran, 2-ethyl-5-methyl-	✓		✓	✓	✓
		2-Furanmethanol, tetrahydro-			✓		✓

Supplementary Table S2. Extended results for GCMS lipid for all evaluated samples. In **bold** are the Odd carbon chain fatty acids, in *italics* the Pinaceae family-markers and ✓ indicate the presence of the molecules.

Chemical class	Carbon chain lenght	Annotated Compound	Inner coffin of Kha (Suppl.8429)	Middle coffin of Kha (Suppl. 8316/01)	Outer bandages of Kha (Suppl.8431)	Inner coffin of Merit (Suppl.8470)	New Kingdom jar (Cat. 3570)
FATTY ACIDS	C2-OH	Glycolic acid, 2TMS derivative			✓		
	C3-OH	Lactic Acid, 2TMS derivative	✓	✓	✓	✓	
	C5:1	4-Pentenoic acid, TMS derivative			✓		
	C5+1	4-Methylvaleric acid, TMS derivative					✓
	C5oxo	Levulinic acid, TMS derivative			✓		
	C6	Hexanoic acid, TMS derivative	✓	✓		✓	
	C6-OH	6-Hydroxyhexanoic acid, 2TMS derivative			✓		
	**C7**	**Heptanoic acid, TMS derivative**	✓	✓			
	C7-OH	7-Hydroxyheptanoic acid, 2TMS derivative			✓		✓
	C8	Octanoic acid, TMS derivative	✓	✓	✓	✓	
	C8-OH	2-Hydroxyoctanoic acid, 2TMS derivative			✓		✓
	C8:oxo	7-Oxooctanoic acid, TMS derivative			✓		✓
	**C9**	**Nonanoic acid, TMS derivative**	✓	✓	✓	✓	✓
	C10	Decanoic acid, TMS derivative	✓	✓	✓	✓	✓
	C10:1	10-Undecenoic acid, TMS derivative			✓		
	**C11**	**Undecanoic acid, TMS derivative**	✓	✓		✓	
	C12	Dodecanoic acid, TMS derivative	✓	✓	✓		✓
	**C13**	**Tridecanoic acid, TMS derivative**	✓		✓	✓	✓
	C14	Myristic acid, TMS derivative	✓	✓	✓	✓	✓
	**C15**	**Pentadecanoic acid, TMS derivative**	✓	✓	✓	✓	✓
	C16	Palmitic Acid, TMS derivative	✓	✓	✓	✓	✓
	C16:1	Palmitoleic acid, TMS derivative		✓	✓		
	**C17**	**Heptadecanoic acid, TMS derivative**	✓	✓	✓		✓
	C18	Stearic acid, TMS derivative	✓	✓	✓	✓	✓
	C18:1	Oleic Acid, TMS derivative		✓ + vaccenic	✓		
	C18:2	9,12-Octadecadienoic acid, TMS derivative				✓	
	C18-2*OH	9,18-Dihydroxyoctadecanoic acid, 3TMS derivative			✓		✓
	C20	Arachidic acid, TMS derivative			✓		✓
	C20:1	11-Eicosenoic acid,TMS derivative			✓		
	C22	Behenic acid, TMS derivative			✓		✓
	C24	Lignoceric acid, TMS derivative			✓		✓
α,ω-CARBOXYLIC ACIDS	C4	Butanedioic acid, 2TMS derivative	✓	✓	✓		✓
	C4:1	2-Butenedioic acid, 2TMS derivative	✓		✓		
	C4+1	Methylsuccinic acid, 2TMS derivative	✓		✓		
	C4+2	2,2-Dimethylsuccinic acid, 2TMS derivative	✓				
	C4-2*OH	Malic acid, 2TMS derivative			✓		
	C5	Pentanedioic acid, 2TMS derivative	✓		✓		✓
	C5+1	2-Methylglutaric acid, 2TMS derivative			✓		
	C5+2	3,3-Dimethylglutaric acid, 2TMS derivative	✓		✓		
	C5+3	Pentanedioic acid, 2,2,3-trimethyl-, 2TMS derivative			✓		
	C6	Hexanedioic acid, 2TMS derivative	✓		✓		✓
	C6+1	3-Methyladipic acid, 2TMS derivative			✓		
	C7	Pimelic acid, 2TMS derivative	✓		✓		✓
	C8	Suberic acid, 2TMS derivative	✓		✓	✓	✓
	C8-Ph	Terephthalic acid, 2TMS derivative	✓	✓			
	C9	Azelaic acid, 2TMS derivative	✓		✓	✓	✓
	C10	Sebacic acid, 2TMS derivative	✓		✓		✓
	C11	Undecanedioic acid, TMS derivative	✓		✓		✓
	C12	Dodecanedioic acid, 2TMS derivative					✓
ALCOHOLS	C3	1-propanol, TMS derivative			✓		
	C4	1,2,3-Butanetriol, 3TMS derivative			✓		
	C4	1,4-Butanediol, 2TMS derivative			✓		
	C10	2-Decanol, TMS derivative					✓
ALKANES	C9	Nonane	✓			✓	✓
	C7	Pentane, 2,3-dimethyl-	✓				
	C8	Pentane, 2,2,3-trimethyl-//Pentane, 2,2,4-trimethyl-	✓		✓		
	C8	Hexane, 2,2-dimethyl- (octane isomer)					✓
	C13	Dodecane, 2-methyl-				✓	
	C13	Tridecane				✓	✓
	C14	Tetradecane				✓	
	C16	Hexadecane			✓		
BENZOIC ACIDS and DERIVATIVES		4-Hydroxybenzaldehyde, TMS derivative			✓		
		4 or 3-Hydroxybenzoic acid, 2TMS derivative	✓	✓	✓	✓	
		4-isopropylbenzoic acid, TMS derivative		✓			
		4-Oxohexanoic acid, TMS derivative			✓		
		Monoallyl phthalate, TMS derivative			✓		
		Protocatechuic acid, 3TMS derivative	✓			✓	
		p-Toluic acid, TMS derivative	✓	✓		✓	
		4-propylbenzoate, TMS derivative	✓			✓	
		2-acetoxy-4-methylbenzoate, TMS derivative			✓		
		Vanillic Acid, 2TMS derivative	✓	✓	✓	✓	
CARBOXYLIC ACIDS and DERIVATIVES		Phenylsuccinic acid, 2TMS derivative			✓		
		3-Methyl-1-cyclohexanecarboxylic acid, TMS derivative					✓
		3-(5-Oxooxolan-2-yl)propanoic acid, TMS derivative					✓
		4-Pyrimidinecarboxylic acid, 1,2,3,6-tetrahydro-2,6-dioxo-1,3-bis(TMS)-, TMS derivative			✓		
		Butanedioic acid, methyl-, bis(1-methylpropyl) ester			✓		
		Cyclohexanol, 3,3,5-trimethyl-, acetate, cis-		✓			
		Propanoic acid, butyl ester	✓				
		Propanoic acid, 2,2-dimethyl-, methyl ester				✓	
		Methyl 9,10-dihydroxystearate, 2TMS derivative			✓		
		Formamide, N,N-diethyl-			✓	✓	
Benzene and substituted derivatives		2-Methyl-6-tert-butylphenol, TMS derivative			✓		
Benzene and substituted derivatives		2-tert-Butyl-6-methylphenol, TMS derivative	✓				
Dioxolanes		1,3-Dioxolane-4-methanol, 2-ethenyl-			✓		
Ethers		diethylene glicole		✓			
Fatty amide		Octadecanamide			✓		
Lactones		7,9-Di-tert-butyl-1-oxaspiro(4,5)deca-6,9-diene-2,8-dione				✓	✓
Lactones		Altronic acid, γ-lactone, 4TMS derivavative			✓		
Lactones		Erythrono-1,4-lactone, (E)-, 2TMS derivative			✓		
Lactones		Hexonic acid, 3-deoxy-, γ-lactone, 2,5,6-tris-O-TMS			✓		
Glycerolipids		Glycerol, 2TMS derivative			✓		✓
Glycerolipids		Glycerol, 1-tert-butyl 3-TMS derivative			✓		
MG(0:0/15:0/0:0)		Pentadecanoic acid, 2-hydroxy-1-(hydroxymethyl)ethyl ester			✓		
MG(0:0/16:0/0:0)		1-Monopalmitin, 2TMS derivative		✓		✓	✓
Monolignols		p-coumaric alcohol			✓		
Abietane diterpenoid		*Dehydroabietic acid, TMS derivative*		✓			
Monoterpenoids		Benzenebutanal, .gamma..4-dimethyl-	✓	✓		✓	
Monoterpenoids		Menthyl valerate = Butanoic acid, 3-methyl-, 5-methyl-2-(1-methylethyl)cyclohexyl ester, (1α,2β,5α)-		✓			
Pentacyclic triterpenoid		Oleanolic acid 2TMS derivative // Ursolic acid 2TMS derivative	✓	✓			
Sesquiterpenoid		Dihydro-ar-turmerone		✓			
Sesquiterpenoids		Cyclopentanone, 3,3,4-trimethyl-4-(4-methylphenyl)-	✓	✓		✓	
Steroids and steroid derivatives		7α-Hydroxyandrost-4-ene-3,17-dione, TMS derivative// 19-Norethandrolone		✓			
Steroids and steroid derivatives		Androst-7-ene, (5α)-		✓			
Sugar		α-D-Mannopyranose, 5TMS derivative			✓		
Sugar		β-L-(-)-Fucopyranose, (R,S,R,R,S)-, 4TMS derivative			✓		
Sugar		D-Mannitol, 6TMS derivative			✓		
Tetrahydrofurans		2(3H)-Furanone, 5-dodecyldihydro-					✓
Thioethers		Propane, 2-methyl-2-[(1-methylethyl)thio]-				✓	
Triol molecule		Glyceric acid, 3TMS derivative			✓		

Inner coffin of Kha (Suppl.8429) | Middle coffin of Kha (Suppl. 8316/01) | Outer bandages of Kha (Suppl.8431) | Inner coffin of Merit (Suppl.8470) | New Kingdom jar (Cat. 3570)

Supplementary Table S3. Extended results for LC-MS lipid for all evaluated samples. In **bold** are the Fatty Acids, in *italics* the Pinaceae family-markers and n.s. means not specific of any plant. *: possible contamination from exte

Inner coffin of Kha (Suppl.8429)

retention time (min)	MONOISOTOPIC MASS		PUTATIVE IDENTIFICATION SOURCE	POSSIBLE SOURCE
3,45	300,0997	Farrerol	mzCloud 98	Rhododendron dauricum, Rhododendron farrerae
3,62	214,1357	7-Hydroxycadalene	mzCloud 82	*n.s.*
3,71	218,1671	(+)-Nootkatone	ChemSpider	Citrus, Cupressaceae
3,82	216,1515	Turmerone	ChemSpider	Curcuma
4,43	*314,1882*	*7-Oxodehydroabietic acid*	*mzCloud 99*	*Pinaceae, Cupressaceae_Coniferous resin*
5,07	456,3601	Oleanolic acid	ChemSpider	Pistacia resin
5,24	486,3344	Quillaic Acid	ChemSpider	*n.s.*
5,29	470,3394	18-β-Glycyrrhetinic acid	ChemSpider, mzCloud 80	Liquorice
6,19	512,3500	3-Acetyl-11-keto-β-boswellic acid	ChemSpider	Boswellia sacra (frankincense)
8,01	438,3497	3-Oxolup-20(29)-en-28-al	mzCloud 97	*n.s.*
8,51	454,3445	Betulonic acid	mzCloud 87	*n.s.*
12,67	339,3467	Docosanamide	mzCloud 93	*n.s.*

Middle coffin of Kha (Suppl. 8316/01)

retention time (min)	MONOISOTOPIC MASS	ANNOTATED COMPOUND	PUTATIVE IDENTIFICATION SOURCE	POSSIBLE SOURCE
1,94	262,1204	Helenalin = 4-hydroxy-6,9-dimethyl-3-methylidene-2H,3H,3aH,4H,5H,6H,6aH,7H,8H,9bH-azuleno[4,5-b]furan-2,8-dione	ChemSpider	*n.s.*
2,48	346,2507	Ginkgoic acid	mzCloud 83	*n.s.*
3,65	214,1357	7-Hydroxycadalene	ChemSpider	*n.s.*
3,84	414,2769	Lupulone	ChemSpider	Hop
4,16	219,1743	(+)-Nootkatone	ChemSpider	Citrus, Cupressaceae
4,44	*314,1882*	*7-Oxodehydroabietic acid*	*mzCloud 99*	*Pinaceae, Cupressaceae_Coniferous resin*
4,77	456,3603	Ursolic acid	mzCloud 81	Dammar resin
4,84	468,3240	3-Oxoglycyrrhetic acid	ChemSpider	Liquorice
5,02	486,3345	Quillaic acid	mzCloud 81	*n.s.*
5,28	466,2718	Wilforol A	ChemSpider	*n.s.*
5,29	470,3395	18-β-Glycyrrhetinic acid	ChemSpider_mzCloud 87	Liquorice
5,65	456,3601	Oleanolic acid	mzCloud 88	Pistacia resin
6,65	436,2977	Bolmantalate	ChemSpider	*n.s.*
6,94	424,3340	α-Tocotrienol	MassList	*n.s.*
7,88	440,3653	Oleanonic aldehyde	mzCloud 97	Dammar resin, Mastic resin
8,08	454,3444	Betulonic acid	mzCloud 87	*n.s.*

Outer bandages of Kha (Suppl.8431)

retention time (min)	MONOISOTOPIC MASS	ANNOTATED COMPOUND	PUTATIVE IDENTIFICATION SOURCE	POSSIBLE SOURCE
0,86	261,2303	Tetradecaphytosphingosine	ChemSpider	Human metabolome
0,98	289,2616	C16 phytosphingosine	ChemSpider	Saccharomyces cerevisiae metabolite*
1,31	176,0836	Ethyl cinnamate	ChemSpider	Cannella/Cinnamomum verum
1,78	248,1411	(3aR,8R,8aR,9aR)-8-Hydroxy-8a-methyl-3,5-bis(methylene)decahydronaphtho[2,3-	mzCloud 81	Stevia
1,92	262,1204	Helenalin	ChemSpider	Arnica montana, Arnica longifolia
2,95	276,1723	Shogaol	mzCloud 90	Zingiber officinalis
**3,11**	**296,2349**	**9-HODE**	**mzCloud 95**	**Metabolite of Mus musculus, Valeriana fauriei, Homo sapiens.***
**3,26**	**278,2244**	**Linolenelaidic acid**	**mzCloud 85**	**Primrose oil, Borage oil, Fungi, Salvia, Tabacco***
3,42	492,2735	Sordarin	ChemSpider	Parascedosporium putredinis (Fungi)*
3,81	321,1938	Croomine	ChemSpider	Croomia japonica
3,87	506,2889	Mucronine A	ChemSpider	Ziziphus mucronata
4,44	*314,1879*	*7-Oxodehydroabietic acid*	*mzCloud 99*	*Pinaceae, Cupressaceae_Coniferous resin*
4,53	262,2296	2,4,6-Tri-tert-butylphenol	mzCloud 97	*n.s.*
**4,53**	**316,2612**	**9,10-Dihydroxystearic acid**	**ChemSpider**	**Oxidation product of Oleic Acid (from vegetable/animal oils & fats)**
**4,78**	**270,2194**	**3-oxopalmitic acid**	**mzCloud 98**	**Human metabolome***
**5,05**	**278,2244**	**Pinolenic acid**	**mzCloud 76**	**Larix, Cedrus**
**5,79**	**296,2349**	**13-Hydroxyoctadecadienoic acid**	**ChemSpider, mzCloud 85**	**Human metabolome***
**5,94**	**298,2505**	**Ricinoleic acid**	**mzCloud 99**	**Ricinus communis**
7,21	255,2561	Palmitamide	ChemSpider	Metabolite of Mus musculus*
9,01	403,3083	N-palmitoyl phenylalanine	ChemSpider	Human metabolome*
9,05	222,0679	Isoflavone	ChemSpider	Pueraria montana, Astragalus membranaceus, Iris domestica
**13,96**	**536,4799**	**FAHFA(16:1/18:0)_5-POHSA**	**ChemSpider**	**Human metabolome***
16,34	565,5430	Ceramide (18:1/18:0)	ChemSpider	Human metabolome*
**16,56**	**282,2557**	**Oleic acid**	**mzCloud 99**	**Vegetable and animal oils and fats**
17,62	699,6370	Ceramide D (C24)	ChemSpider	Saccharomyces cerevisiae metabolite*
18,34	683,6423	N-(2-hydroxytetracosanoyl)phytosphingosine	ChemSpider	Sphingolipid human metabolome*
18,69	727,6685	N-(2,3-dihydroxyhexacosanoyl)-(4S)-hydroxysphinganine	ChemSpider	Saccharomyces cerevisiae metabolite*
19,43	711,6735	N-(2-hydroxyhexacosanyl)-(4R)-phytosphingosine	ChemSpider	Sphingolipid human metabolome*

Inner coffin of Merit (Suppl.8470)

retention time (min)	MONOISOTOPIC MASS	ANNOTATED COMPOUND	PUTATIVE IDENTIFICATION SOURCE	POSSIBLE SOURCE
0,86	261,2303	Tetradecaphytosphingosine	ChemSpider	*n.s.*
0,98	289,2616	C16 phytosphingosine	ChemSpider	Saccharomices cerevisiae metabolome*
1,29	243,1258	Agomelatine	ChemSpider	Human metabolome*
2,13	174,0680	Menadiol	ChemSpider, mzCloud 82	E. Coli metabolome*
**2,29**	**201,1728**	**11-aminoundecanoic acid**	**ChemSpider, MassList**	***n.s.***
**3,55**	**229,2040**	**2-aminotridecanoic acid**	**ChemSpider**	**Metabolite of Mus musculus***
3,66	202,1356	Jasmonal	ChemSpider	*n.s.*
3,82	216,1513	Turmerone	ChemSpider	Curcuma
4,16	218,167	(+)-Nootkatone	ChemSpider	Citrus, Cupressaceae
4,43	*314,188*	*7-oxodehydroabietic acid*	*mzCloud 99*	*Pinaceae, Cupressaceae_Coniferous resin*
5,48	470,3394	18-β-Glycyrrhetinic acid	ChemSpider, mzCloud 82	Licorice
5,54	454,3445	Masticadienonic acid	MassList, mzCloud 78	Mastic resin
5,56	450,3132	Dysolenticin B	ChemSpider	Dysoxylum lenticellatum
8,02	454,3444	Moronic acid	ChemSpider, mzCloud 87	Mastic resin
8,14	440,3650	β-Amyrenonol	mzCloud 93	*n.s.*
9,19	440,3650	Betulinicaldehyde	mzCloud 88	*n.s.*
12,75	339,3499	Docosanamide	mzCloud 93	*n.s.*

New Kingdom jar (Cat. 3570)

retention time (min)	MONOISOTOPIC MASS	ANNOTATED COMPOUND	PUTATIVE IDENTIFICATION SOURCE	POSSIBLE SOURCE
0,99	289,2615	C16 phytosphingosine	ChemSpider	Saccharomices cerevisiae metabolome*
1,54	404,2044	Lauroside B	ChemSpider	Laurus nobilis
**3,01**	**296,2350**	**13-Hydroxyoctadecadienoic acid**	**mzCloud 92**	**Ricinus communis/oxidation product of Linoleic Acid (from vegetable/animal oils & fats)**
**4,28**	**316,2612**	**9,10-Dihydroxystearic acid**	**ChemSpider**	**Oxidation product of Oleic Acid (from vegetable/animal oils & fats)**
4,28	340,2974	Glycidyl Stearate	ChemSpider	*n.s.*
**4,28**	**298,2473**	**Ricinoleic Acid**	**mzCloud 92**	**Ricinus communis**
4,78	224,2139	Dihydrofarnesol	ChemSpider	*n.s.*
**5,61**	**322,2506**	**15-oxo-11,13-eicosadienoic acid**	**mzCloud 82**	***n.s.***
**9,05**	**256,2401**	**Palmitic acid**	**ChemSpider, MassList**	**vegetable/animal oils & fats**

Supplementary Table S4. Extracted CSV.file from original AcquireX sequence file.

LINE 1	Bracket Type=4,Sequence Capabilites=ReInject
LINE 2	Sample Type,File Name,Sample ID,Path,Instrument Method,Process Method,Calibration File,Position,Inj Vol,Level,Sample Wt,Sample Vol,ISTD Amt,Dil Factor,L1 ,L2 ,L3 ,L4 ,L5 ,Comment,Sample Name,
LINE 3	**Blank**,Solvent_01,1,D:\PROJECTS\MUMMIES_Eurac\2024\campione 2_\ReBi_2024_campione2,D:\PROJECTS\MUMMIES_Eurac\2024\campione 2_\ReBi_2024_campione2\LIPID_C18_APCI_AQX_FS_statistico,,,B:C2,2,,0,0,0,1,,,,,,,Solvent_01,
LINE 4	**Blank**,Blank_01,1,D:\PROJECTS\MUMMIES_Eurac\2024\campione 2_\ReBi_2024_campione2,D:\PROJECTS\MUMMIES_Eurac\2024\campione 2_\ReBi_2024_campione2\LIPID_C18_APCI_AQX_FS_statistico,,,B:D2,2,,0,0,0,1,,,,,,,Blank_01,
LINE 5	**Std Bracket**,Blank_02,1,D:\PROJECTS\MUMMIES_Eurac\2024\campione 2_\ReBi_2024_campione2,D:\PROJECTS\MUMMIES_Eurac\2024\campione 2_\ReBi_2024_campione2\LIPID_C18_APCI_AQX_FS_statistico,,,B:D2,2,,0,0,0,1,,,,,,,Blank_02,
LINE 6	QC,**ReBi_2024_campione2**,1,D:\PROJECTS\MUMMIES_Eurac\2024\campione 2_\ReBi_2024_campione2,D:\PROJECTS\MUMMIES_Eurac\2024\campione 2_\ReBi_2024_campione2\LIPID_C18_APCI_AQX_FS_statistico,,,B:E2,2,,0,0,0,1,,,,,,,ReBi_2024_campione2,
LINE 7	Unknown,**ID_campione2_01**,ID 1,D:\PROJECTS\MUMMIES_Eurac\2024\campione 2_\ReBi_2024_campione2,D:\PROJECTS\MUMMIES_Eurac\2024\campione 2_\ReBi_2024_campione2\LIPID_C18_APCI_AQX_Excl_Incl_01,,,B:E2,2,,0,0,0,1,,,,,,,ID_campione2_01,
LINE 8	Unknown,**ID_campione2_02**,ID 2,D:\PROJECTS\MUMMIES_Eurac\2024\campione 2_\ReBi_2024_campione2,D:\PROJECTS\MUMMIES_Eurac\2024\campione 2_\ReBi_2024_campione2\LIPID_C18_APCI_AQX_Excl_Incl_02,,,B:E2,2,,0,0,0,1,,,,,,,ID_campione2_02,
LINE 9	Unknown,**ID_campione2_03**,ID 3,D:\PROJECTS\MUMMIES_Eurac\2024\campione 2_\ReBi_2024_campione2,D:\PROJECTS\MUMMIES_Eurac\2024\campione 2_\ReBi_2024_campione2\LIPID_C18_APCI_AQX_Excl_Incl_03,,,B:E2,2,,0,0,0,1,,,,,,,ID_campione2_03,
LINE 10	Unknown,**ID_campione2_04**,ID 4,D:\PROJECTS\MUMMIES_Eurac\2024\campione 2_\ReBi_2024_campione2,D:\PROJECTS\MUMMIES_Eurac\2024\campione 2_\ReBi_2024_campione2\LIPID_C18_APCI_AQX_Excl_Incl_04,,,B:E2,2,,0,0,0,1,,,,,,,ID_campione2_04,
LINE 11	**Blank**,,,D:\PROJECTS\MUMMIES_Eurac\2024\campione 2_\ReBi_2024_campione2,D:\PROJECTS\MUMMIES_Eurac\2024\campione 2_\ReBi_2024_campione2\LIPID_C18_APCI_AQX_Excl_Incl_04,,,B:E2,2,,0,0,0,1,,,,,,,ID_campione2_04,

**LEGEND**	
LINE 1	*Authomatic info about method*
LINE 2	*Authomatic info about sample list description*
LINE 3	SAMPLE LIST: **Solvent 01 --> only solvent**
LINE 4	SAMPLE LIST: **Blank 01 --> procedural blank**
LINE 5	SAMPLE LIST: **Blank 02 --> procedural blank**
LINE 6	SAMPLE LIST: **Sample 2**
LINE 7	SAMPLE LIST: **1st iterated injection of sample 2**
LINE 8	SAMPLE LIST: **2nd iterated injection of sample 2**
LINE 9	SAMPLE LIST: **3rd iterated injection of sample 2**
LINE 10	SAMPLE LIST: **4th iterated injection of sample 2**
LINE 11	*Authomatic info about method*

Supplementary Table S5. Total amount of each initial sampling and material used for the sample preparation.

	**Inner coffin of Kha** (Suppl.8429)	**Middle coffin of Kha** (Suppl. 8316/01)	**Outer bandages of Kha** (Suppl.8431)	**Inner coffin of Merit** (Suppl.8470)	**New Kingdom jar** (Cat. 3570)
Initial sampling *(mg)*	12,0	4,5	29,7	4,6	11,2
Amount used for GC-MS bitumen *(mg)*	5,1	0,9	0,6	0,8	1,8
Amount used for GC-MS lipid *(mg)*	1,9	1,2	2,0	0,9	1,1
Amount used for LC-HRMS molecular profiling focused on lipids *(mg)*	2,9	0,8	2,2	1,3	1,5
