# Supplementary material for: Characterization of complex New Kingdom funerary resinous mixtures uncovers liquorice-derived substances and pine nut oil
Source: Sci Rep. 2026 Jun 11;16:26611. doi: 10.1038/s41598-026-55926-7 (PMC13503808; doi:10.1038/s41598-026-55926-7)
Supplement: Supplementary file 2 [file 41598_2026_55926_MOESM2_ESM.docx]

**SUPPLEMENTARY INFORMATION**

TEXT S1. Samples selection

1.1 Inner coffin of Kha (Suppl.8429)
1.2 Middle coffin of Kha (Suppl.8316/01)
1.3 Outer bandages of Kha (Suppl.8431)
1.4 Inner coffin of Merit (Suppl.8470)
1.5 New Kingdom small jar (Cat.3570)

TEXT S2. Liquorice enigma

FIGURE S1

REFERENCES

----------

Text S1. Samples selection

The organic samples were collected at the Museo Egizio in Turin (Italy) from five distinct locations: the inner coffin (paragraph 1.1) and the middle coffin (1.2) as well as the outer bandages (1.3) of the mummified male individual Kha. Additionally, a sample was taken from the inner coffin of the mummified female individual Merit (1.4). A separate sample, contemporaneous with Kha and Merit but not associated with their grave goods, was also collected from a New Kingdom (NK) small jar (1.5).

The Museo Egizio systematically applies the UNI EN 16085:2012^1^ standard to all sampling operations involving cultural heritage items. All samples collected within the framework of this study, none of which included human remains, were obtained in accordance with this regulation, which establishes standardized methodologies and strict criteria for the sampling of cultural heritage materials. As required by Article 21 of Legislative Decree 42/2004 (*Codice dei beni culturali e del paesaggio*), official authorization was duly requested and obtained from the competent local office of the Italian Ministry of Cultural Heritage. No institutional ethical review was required for this study.

The exact sampling locations and descriptions of the collected samples are provided in the tables presented in the following paragraphs.

1.1 Inner coffin of Kha (Suppl.8429)

In 2017, in the framework of a conjoint interdisciplinary project between Eurac Research and the Museo Egizio in Turin^2^, a few milligrams of a black, sticky and pulverised substance accumulated inside the inner coffin were collected.

Kha, a prominent figure in Theban society, commissioned a set of nested wooden coffins. The innermost coffin, made of wood from the Upper Nile, contained Kha’s human remains and was adorned with garlands of flowers and leaves^3^. This coffin closely replicates the middle one in both its anthropomorphic shape and the funerary texts inscribed on its exterior. However, it is distinguished by a gold leaf covering, applied over a fine layer of gypsum, symbolizing the deceased’s assimilation into the solar regenerative cycle^4^.

| *Inventory No.* | **Suppl.8429** | |
| --- | --- | --- |
|  | | |
| *The sampling location* | Inside the coffin, in the bottom part. | |
| *Description of the sample* | Dark-colored surface layer, small pieces located at the base of the coffin. | |
| *Images of the sampling phase (left) and of the sample (right)* |  |  |
| *Sampling method* | Tweezers. | |

1.2 Middle coffin of Kha (Suppl.8316/01)

The intermediate coffin, made of wood, *Pistacia* sp. resin^5^ and gold leaf, replicates the anthropomorphic features of the deceased. It is adorned with a funerary mask featuring inlaid eyes of glass paste and alabaster, as well as a broad *usekh* collar, which is covered with a garland of melilot intertwined with *Centaurea depressa* flowers and lotus petals^3^. The combination of black and gold symbolizes the duality of the sun and the darkness of the afterlife, reflecting the regeneration of the sun god Ra through the transformative power of Osiris^4^.

| *Inventory No.* | **Suppl.8316/01** | |
| --- | --- | --- |
|  | | |
| *The sampling location* | Left side, isolated, above the centre text panel. | |
| *Description of the sample* | Dark-colored surface layer. | |
| *Images of the sampling phase (left) and of the sample (right)* |  |  |
| *Sampling method* | Scalpel. | |

1.3 Outer bandages of Kha (Suppl.8431)

The body of Kha was wrapped in multiple layers of high-quality bandages and covered with an external shroud. Additional outer bandages were arranged vertically from head to foot to secure the shroud, while four tighter bands were applied to stabilize the upper and lower limbs.^3^ In some areas, a non-uniform application of dark embalming substance is observed. Its precise functional role remains difficult to define; however, it may represent: (i) an embalming balm originally applied to the body and/or wrappings and/or (ii) substances used during subsequent funerary ritual actions, such as anointing or libations.

Sampling locations were selected in consultation with museum conservators, avoiding visibly restored or heavily handled areas, and conservation records indicate no documented use of modern consolidants or solvent-based treatments at the sampled spots. No chemical cleaning (e.g., solvents, detergents) or mechanical decontamination (e.g., abrasion, scraping of outer layers) was applied prior to analytical extraction, in order to avoid removing or altering the original substance.

| *Inventory No.* | Suppl.8431 | |
| --- | --- | --- |
|  | | |
| *The sampling location* | Left side, thigh level. | |
| *Description of the sample* | Dark-colored surface layer deposited on the textile. | |
| *Images of the sampling phase (left) and of the sample (right)* |  |  |
| *Sampling method* | Scalpel and tweezers. | |

1.4 Inner coffin of Merit (Suppl.8470)

Merit, unlike her husband Kha, had only two coffins, one of which was the inner coffin, which was disproportionately large (maximum length of 199 cm) compared to the mummy (160 cm), with the empty spaces inside padded with linen fabrics in antiquity^4^. One hypothesis suggests that the coffin was initially intended for Kha, but the premature death of his wife necessitated its reuse^6^. The external decoration, which is not uniform and includes texts from Kha's coffin in his name, supports this hypothesis^4^.

| *Inventory No.* | **Suppl.08470** | |
| --- | --- | --- |
|  | | |
| *The sampling location* | Left side, isolated second panel from the feet, upper portion. | |
| *Description of the sample* | Dark-colored surface layer. | |
| *Images of the sampling phase (left) and of the sample (right)* |  |  |
| *Sampling method* | Scalpel and tweezers. | |

1.5 New Kingdom small jar (Cat.3570)

| *Inventory No.* | **Cat.3570** | |
| --- | --- | --- |
|  | | |
| *The sampling location* | Bottom of the jar. Macroscopically, the inner and outer substance form a single, continuous deposit, strongly suggesting that the exterior streaks represent spillage or overflow of the original content rather than an unrelated transfer. | |
| *Description of the sample* | Dark-colored substance. | |
| *Images of the sampling phase (left) and of the sample (right)* |  |  |
| *Sampling method* | Scalpel. | |

This small (height and width around 9.5 cm) jar is made of red clay and features a rounded base, a short neck, and a flared rim with a rounded lip. The exterior of the vessel is covered with strips of linen fabric. Furthermore, the body is decorated with black painted lines forming a pattern of parallel horizontal stripes and little circles.

Although its archaeological context is unknown, its typology and decorative style suggest a Nubian provenance (in present-day southern Egypt and northern Sudan) and a dating to the New Kingdom (1539–1077 BCE)^7^. However, its function remains uncertain, including whether it was used in funerary contexts, embalming practices, or both. Nevertheless, the little jar was included in this study on the basis of its typological characteristics and earlier proposals that similar containers may have been used to hold oily or resinous substances. In addition, the visible presence of a bituminous residue, together with unpublished Pyrolysis–Gas Chromatography/Mass Spectrometry (Py-GC/MS) analyses conducted by the museum, further supports a possible association with a funerary context and with mummification practices.

Text S2. Liquorice enigma

The claim that liquorice (*Glycyrrhiza glabra*) was used in ancient Egypt has been critically assessed and found to lack credible support in current Egyptological research. Major botanical and medical texts from ancient Egypt^8,9^ contain no verified references to liquorice, and authoritative databases^10,11^ list no matching terms. Moreover, there is a notable absence of corresponding terminology in ancient Egyptian and Coptic sources.

The use of liquorice in ancient Egypt is linked by Non-Egyptologists to the NK Papyrus Ebers^12,13^ (1514-1494 BCE), but there is no direct evidence to support these claim. The term "bḥḥ"^14^ which appears in the [Ebers 264 (49.5)](https://thesaurus-linguae-aegyptiae.de/sentence/IBYBYKysQ0CQOUKIuQWDW66RgaM)^15^ (mny.t n.t bḥḥ) remains botanically unidentified. While "mny."^15^ is sometimes translated as "root" this interpretation is not firmly established. Additionally, the word for "sweet" appears in a recipe in the Ebers Papyrus, but it refers to sweet beer, not to liquorice, and does not offer a convincing connection to liquorice in this context. Therefore, the accurate of translation of Ebers 264 (49.4-6) would be: “Another (remedy) for regulating excessive urination (?): gw-grass: 1 dose; berries of the Phoenician juniper: 1 dose; root of the bḥḥ-plant: 1 dose. Pound into a homogeneous mass; let stand overnight with sweet beer. Drink it, including the sediment (?).”

In the scholarly literature, there are no references to *Glycyrrhiza glabra* in the works of Egyptologists neither in the Grundriss der Medizin der alten Ägypter^16^. Notably, major scholars, including Lucas^17^, Keimer^18^, and Germer^19^, do not mention liquorice in their works on ancient Egyptian botanical or medical practices. Manniche^20^ does include an entry on liquorice, but it only discusses its use in classical antiquity and states explicitly at the very end: “Considering this wide use it is surprising that no word for liquorice has yet been identified in Coptic or ancient Egyptian medical texts.”

The Roman period *Papyrus London-Leiden* 24,8 (200-225 AD), which contains a later recipe, includes a term that resembles the Greek word "melakretikou" (possibly from *melikretos*, meaning “honey-water”), but this is not definitively identified as liquorice and is not from the pharaonic period^[1]^.

The alleged presence of liquorice in the tomb of Tutankhamun^21^ is unsubstantiated, and claims in popular sources appear to be the result of "progressive embellishment". Modern misunderstandings, such as the idea that *mai sus* (an Arabic name for liquorice water) was an ancient Egyptian drink, stem from a misapplication of contemporary Egyptian practices to the ancient period. Modern misconceptions—such as the belief that *mai sus* (an Arabic term for liquorice water) was consumed in ancient Egypt—arise from projecting contemporary Egyptian customs onto the ancient past. In reality, *mai sus* refers to a modern beverage^22^, not one from antiquity. As noted by Manniche^20^, *sus* is the Arabic word for liquorice, and it was also known in Akkadian as *šūšū*, indicating a likely Semitic origin. The term *mai* is almost certainly derived from the Arabic *maya*, meaning “water”.

The following conclusions can be drawn from the available evidence:

1. There is no conclusive evidence for the identification of liquorice in ancient Egyptian texts or finds.
2. The botanical term "bḥḥ" in the Ebers Papyrus (49.5) remains unidentified and is not convincingly interpreted as *Glycyrrhiza glabra*.
3. Previous claims regarding the use of liquorice in ancient Egypt are not based on scientific evidence but rather on speculation, misunderstandings, or the uncritical adoption of secondary sources.
4. The chemical traces found in samples from the tomb of Kha and Merit may represent the earliest archaeochemical evidence of the plant in an Egyptian context.

In conclusion, while the historical use of *Glycyrrhiza glabra* in ancient Egyptian remains unverified due to the absence of textual or iconographic references, the consistent chemical identification of its key molecular markers, 18-β-glycyrrhetinic acid and 3-oxo-glycyrrhetinic acid, provides biomolecular evidence for its presence.

This finding represents a potentially significant contribution to our understanding of New Kingdom embalming practices and underscores the value of molecular research in revealing aspects of ancient rituals that may not be preserved in material culture and the written record.

**Figure S1.** Experimentally obtained mass spectrometric data, used for the identification of 18-β-Glycyrrhetinic Acid (18-β-GA) which is a typical marker of liquorice presence. The data shown are from sample Suppl.8316/01, analyzed with negative ionization mode. **a)** Extracted ion chromatogram of MW (Molecular Weight) 470.33939, with chromatographic peak of 18-β-GA at RT=5.360 min. **b)** MS1 spectrum of m/z 469.33209 acquired at RT=5.360 min. **c)** m/z 469.33148 peak of the associated ID1 scan, aligned with (a). **d)** MS2 spectra of parent ion m/z 469.3315@30. **e)** mirror plot comparing experimental MS2 mass spectrum acquired and library MS2 spectrum from mzCloud.

REFERENCES

1.	UNI EN 16085:2012 Conservazione dei beni culturali - Metodologia per il campionamento dei materiali costituenti i beni culturali - Regole generali.

2.	Samadelli, M. *et al.* Development of passive controlled atmosphere display cases for the conservation of cultural assets. *ELSEVIER Fr.-Ed. Sci. MEDICALES ELSEVIER* **35**, 9 (2019).

3.	Marochetti, E. F. *MUMMIE EGIZIE DI TORINO*. vol. 2 (Franco Cosimo Panini Editore S.p.A, 2023).

4.	Ferraris, E. *La Tomba Di Kha e Merit*. (Franco Cosimo Panini Editore S.p.A, 2018).

5.	Bianucci, R. *et al.* Shedding New Light on the 18th Dynasty Mummies of the Royal Architect Kha and His Spouse Merit. *PLOS ONE* **10**, (2015).

6.	Greco, C. the Discovery and the Gravegoods of the Tomb of Kha and Merit. in *Mummies. Interdisciplinary dialogues* (eds Harari, M. & Malgora, S.) (Pavia University Press, 2024).

7.	Holthoer, R. *New Kingdom Pharaonic Sites: The Pottery*. vol. v. 5,1 (Scandinavian University Books, 1977).

8.	Wolfhart, W. *Handbuch Der Altägyptischen Medizin*. (1999).

9.	Dils, P. Thesaurus Linguae Aegyptiae. *Thesaurus Linguae Aegyptiae* https://thesaurus-linguae-aegyptiae.de/object/HTRIEAWSWRDEXA64SRMW7C5WJU.

10.	Thesaurus Linguae Aegyptiae. https://thesaurus-linguae-aegyptiae.de/home.

11.	VÉgA- Vocabulary of Ancient Egyptian. https://vega-vocabulaire-egyptien-ancien.fr/en/welcome-page/.

12.	Ebers Papyrus. https://bxscience.edu/ourpages/auto/2008/11/10/43216077/egypt%20medicine.pdf.

13.	Shibata, S. A Drug Over Millennia: Pharmacognosy, Chemistry and Pharmacology of Licorice. *Pharm. Soc. Jpn.* **120**, (2000).

14.	Thesaurus Linguae Aegyptiae. https://thesaurus-linguae-aegyptiae.de/lemma/56880.

15.	Popko, L. Thesaurus Linguae Aegyptiae. https://thesaurus-linguae-aegyptiae.de/sentence/IBYBYKysQ0CQOUKIuQWDW66RgaM.

16.	von Deines, H. & Grapow, H. *Wörterbuch der Ägyptischen Drogennamen, Grundriss der Medizin der alten Ägypter*. vol. Vol. VI (De Gruyter, Berlin, 1959).

17.	Lucas, A. *Ancient Egyptian Materials*. (Edward Arnold LTD, London, 1926).

18.	Keimer, L. *Die Gartenpflanzen im Alten Ägypten*. (Berlin, 1924).

19.	Germer, R. *Handbuch der altägyptischen Heilpflanzen*. (2008).

20.	Manniche, L. *An Ancient Egyptian Herbal*. (University of Texas Press, 1989).

21.	Loftin, H. King Tut’s Black Treasure. *Sci. Newsl.* **64**, 90 (1953).

22.	Licorice the Versatile. *Sci. Am.* **141**, 210 (1929).

1. F. Ll. Griffith, H. Thompson, The Demotic Papyrus London and Leiden, London 1904, 1909, 1921. Griffith and Thompson do indeed speculate that line 24.8 might mention “liquorice (?)”, but in a footnote both author wonder whether it might actually be the greek word μελίκρητον, meaning “honey and water”. Vittmann, on the other hand, suggests it might mean “Cretan apples”; see Vittmann, https://thesaurus-linguae-aegyptiae.de/sentence/IBUBd1wcNquSNUJxqgQV3O4Prx4, in: Thesaurus Linguae Aegyptiae (accessed: 5/17/2025)
